# The impact of soluble HLA-G in IVF/ICSI embryo culture medium on implantation success

**DOI:** 10.3389/fimmu.2022.982518

**Published:** 2022-11-24

**Authors:** Paweł Radwan, Agnieszka Tarnowska, Karolina Piekarska, Andrzej Wiśniewski, Rafał Krasiński, Michał Radwan, Izabela Nowak

**Affiliations:** ^1^ Gameta Hospital, Department of Reproductive Medicine, Rzgów, Poland; ^2^ Laboratory of Immunogenetics and Tissue Immunology, Ludwik Hirszfeld Institute of Immunology and Experimental Therapy, Polish Academy of Sciences, Department of Clinical Immunology, Wrocław, Poland

**Keywords:** SHLA-G, *in vitro* fertilization, embryo, reproductive success, ovarian stimulation

## Abstract

The HLA-G molecule is widely accepted as an important factor for pregnancy success. Its expression has been detected in the extravillous trophoblasts. Soluble HLA-G (sHLA-G) was found in the genital tract, pre-implanted embryos as well as in seminal fluid. In this study, we investigated the concentration of sHLA-G (sHLA-G1 and sHLA-G5) in media from 344 single cultured embryos following *in vitro* fertilization/intracytoplasmic sperm injection (IVF/ICSI). The level of sHLA-G (U/ml) was tested with a sandwich enzyme-linked immunosorbent assay (ELISA) kit. We correlated sHLA-G secretion with ovarian stimulation protocols, the type of embryo transfer (fresh or frozen cycle) and the quality of the embryos. The ovarian stimulation protocol affects the secretion of sHLA-G by the embryo. Embryos obtained from the long agonist protocol secreted more sHLA-G than those originating from the short antagonist protocol (p = 0.0001). Embryos whose transfer resulted in a clinical pregnancy and/or live birth secreted more sHLA-G compared to those whose transfer ended without pregnancy. This was particularly observable in embryos following the long ovarian stimulation protocol and from a frozen embryo cycle. In conclusion, sHLA-G secreted by the embryo has an impact on implantation and live birth and could be a developmental potential marker of the embryo. Its concentration depends on the ovarian stimulation protocol used.

## Introduction

HLA-G, one of the non-classical HLA class I molecules, is mainly restricted to expression at the extravillous trophoblast (1) and is widely accepted as a factor in pregnancy success (2, 3). Previously published studies, both by other researchers and our team, indicate that the HLA-G molecule is involved in mechanisms of reproductive immunology even before conception, because HLA-G can be detected in the genital tract (4), and in the blood of non-pregnant women (2) and is also present in the seminal fluid of men (5–7). Moreover, HLA-G expression has been found in the pre-implanted embryo (3, 8). Therefore, it seems very probable that the combined HLA-G participation of the mother, father and their embryo may influence the implantation and the success of the pregnancy.

The gene structure of HLA-G consists of 8 exons and 7 introns. Exon 1 encodes signal peptides whereas exons 2, 3, and 4 encode the extracellular domains α1, α2, and α3, respectively. Exons 5 and 6 encode the transmembrane and cytoplasmic domains. However, a stop codon in exon 6 inhibits the formation of the mature mRNA encoded by exon 7, and thus exon 8 is not translated. So far, seven different isoforms resulting from alternative splicing have been described: four membrane-bound (G1-G4) and three soluble (G5-G7) protein variants (3, 9, 10).

HLA-G was expressed at the mRNA and protein level by human oocytes and preimplantation embryos at each cleavage stage up to the blastocyst (11). Different alternative HLA-G mRNAs in preimplantation embryos have been reported by Yao et al. (8), indicating the truncated HLA-G3 and G4 mRNAs as the predominant spliced transcripts (8). Moreover, HLA-G5 was not found until the morula stage and was poorly expressed in comparison to HLA-G1, which was dominantly detected in blastocysts. In other studies, blastocysts in contact with the endometrium at the implantation site, expressed HLA-G1 and – G5 proteins. After implantation, the HLA-G1, -G2, and -G6 isoforms are expressed in invading cytotrophoblasts rather than in syncytiotrophoblasts. However, HLA-G5 was common in trophoblast subpopulations (12, 13).

Menicucci et al. (14) were the first who reported the presence of non-classical soluble HLA proteins in human oocyte and embryo culture supernatants measured during *in vitro* fertilization (IVF) embryo culture (14). However, the antibodies used in this study were not HLA-G specific. A few years later (in 2002 to be precise) the same group published a study on sHLA-G levels in the media of IVF or intracytoplasmic sperm injection (ICSI) embryos using already specific antibodies in ELISA assays (15). Since then, there have been several studies that indicate rather a positive effect of sHLA-G secreted by embryos on implantation success and pregnancy rates (15–28). It is worth mentioning that shed or proteolytically cleaved HLA-G1 (sHLA-G1) can also potentially influence pregnancy outcomes (29).

In this study, we have investigated sHLA-G (sHLA-G1 and sHLA-G5) concentration in media from single cultured embryos over 24-h time periods up to 6 days after IVF/ICSI. We studied its influence on pregnancy success depending on the cycle used – fresh or frozen. Additionally, we correlated sHLA-G secretion with embryo quality as well as the ovarian stimulation protocols.

## Material and methods

### Patients

Patients were qualified in 2015-2020 at the Gameta Clinic in Rzgów. Characteristics of patients involved in our study are presented in **Table 1**.

**Table 1:** Clinical characteristics of couples participating in IVF-ET.

Aspect	Mean ± SD	Range
Age of woman (N = 72)	32.49 ± 4.35	22-44
Age of partner (N = 72)	34.84 ± 4.71	24-47
Number of all embryos (N = 344)	4.81 ± 2.48	1-12
Number of IVF-ET (N = 103)	1.44 ± 0.96	0-4
Number of transferred embryos (N = 120)	1.68 ± 1.12	0-5
**Indications for IVF**	**Number**	**Percent (%)**
Only male factor	21	29.17
Only female factor	22	30.56
Both factors	6	8.33
Idiopathic	23	31.94

IVF-ET, *in vitro* fertilization embryo-transfer; SD, standard deviation

### Ovarian stimulation protocols

In order to stimulate ovulation, the long agonist protocol or the short antagonist protocol was used. The recombinant FSH (rFSH) or human menopausal gonadotropin (hMG) was administrated at a daily dose of 150-300 IU. In the case of the long protocol, pituitary desensibilization was achieved by the daily administration of a GnRH agonist (0.1 mg Gonapeptyl, Ferring Pharmaceuticals). The growth of follicles was monitored by transvaginal ultrasound examination and the measurement of serum estradiol levels (E2). In females qualified for the short antagonist protocol, the procedure of ovarian stimulation was started on the second day of the cycle. When the mean diameter of one of the follicles exceeded 14 mm or when the estradiol level was above 400 pg/ml, patients were administered 0.25 mg of Ganirelix (Orgalutran, Organon). After the diameter of the follicles was greater than 17 mm and the estradiol level was above 200 pg/ml per one follicle, the patient was administered subcutaneously 250 µg rhCG (Ovitrelle, Merck-Serono). Ovarian pick up (OPU) was performed under general intravenous anesthesia 36 hours following the injection of rhCG.

### Oocyte preparation

The follicular fluid was transferred to Petri dishes (BD Falcon). Cumulus oocyte complexes (COCs) were found and incubated for 2 hours in G-IVF PLUS (Vitrolife, Sweden) at 37°C. The oocytes were denudated by removing granulosa cells from them and rinsing them in 1 ml hyaluronidase for 15 seconds (Fertipro, Belgium). Next, the granulosa cells were mechanically separated in a G-MOPS PLUS medium. The oocytes which achieved the metaphase of the second meiotic division (MII) were incubated in G-1 PLUS (Vitrolife, Sweden) for 1-2 hours.

### Sperm preparation

Sperm preparation was proceeded by centrifugation in GM501 SpermAir Washing Medium (Gynemed, Germany) at 250 g for 10 minutes. Before the ICSI procedure, the spermatozoa were immobilized in a drop of 7% PVP (Polyvinylpyrrolidone, SAGE, USA).

### IVF/ICSI procedure and embryo culture

Intracytoplasmic sperm injection (ICSI) was performed with micromanipulators (Eppendorf, Germany) and an inverted microscope (Leica DMI 3000B, Germany). Spermatozoa were microinjected under a 200 x magnitude in a drop of G-MOPS PLUS (Vitrolife, Sweden). Fertilized oocytes were placed separately in 20 µl droplets of G-1 PLUS Medium in Microdroplets Culture Dish (Vitrolife). Zygotes and embryos were cultured in sequential media (G-1 PLUS/G-2 PLUS, Vitrolife, Sweden). The culture was conducted in standard low oxygen conditions (6% CO_2_ and 5% O_2_, 37°C) using HeraCell 150 incubators (Thermoscientific, Germany). On day 3 the G-1 PLUS Medium was changed to the G-2 PLUS Medium. 15 µl of G-1 PLUS Medium from each droplet was stored at -70°C.

### Endometrial preparation and frozen embryo transfer

Estradiol (Estrofem 2 mg, Novo Nordisk) was administered orally, starting on the second day of the target cycle with a dosage of 6 mg/day for endometrial preparation. An ultrasound examination was performed to assess endometrial thickness and pattern 10-12 days following estradiol initiation. Endometrial thickness was measured and recorded as the maximum distance (mm) between the myometrium and the endometrial surface. A thickness of ≥ 7.5 mm was considered satisfactory for initiation of progesterone supplementation. In order to supplement the luteal phase, patients were administered intravaginally 2 x 400 mg micronized progesterone daily. Additionally, oral dydrogesterone (Mylan Healthcare) 3 x 10 mg daily was administrated. Blastocysts were transferred on day 5 or day 6 following progesterone administration (depending on the developmental stage).

### Embryo assessment

The zygotes were assessed on the basis of two pronuclei and two polar bodies in the perivitelline space 16-18 hours following the application of the IVF/ICSI procedure. On days: 2 and 3 (43-45 and 67-69 hours following the microinjection, respectively) the embryos were evaluated in terms of the number, size, symmetry of blastomeres, multinucleation and degree of fragmentation (grade A, B, C, D) – **Supplementary Table 1**. On the fourth day, the embryo was assessed by confirming the presence or absence of morula. Blastocysts on day 5 and day 6 (114-118 and 138-142 hours following the IVF/ICSI procedure) were rated on the basis of blastocoel classification and the degree of embryonic expansion. The embryoblast and trophoblast were scored in the expanded blastocyst according to the modified Gardner scoring system (**Supplementary Table 1**) (30, 31).

### Transfer policy

Embryo transfer (ET) was deferred in case of a premature progesterone rise (> 1.4 ng/ml on the day of hCG administration) or risk of Ovarian Hyperstimulation Syndrome (OHSS). All patients had the best possible quality embryo transferred. The day of transfer was scheduled in the cleavage or blastocyst stage (day 5). The remaining embryos underwent the cryopreservation procedure in the blastocyst stage with vitrification (Cryotop Safety Kit, Kitazato, Japan).

### Pregnancy assessment

Ten days following the embryo transfer, the level of serum hCG was measured. If the result was positive (> 5 mIU/ml), thirty days following the transfer the patients underwent a transvaginal ultrasound examination in order to confirm clinical pregnancy (visualization of a one or more gestational sacs). Forty days after the transfer another ultrasound examination was performed to confirm the presence of an ongoing clinical pregnancy (clinical pregnancy with fetal heart beat – FHR). The pregnancies were followed up and the data collected, including miscarriage and live birth.

### Soluble HLA-G assay

We had access to 344 samples of G1 Plus medium (Vitrolife, Sweden) from day 3 of embryo culture. The concentration of sHLA-G (U/ml) in the medium was tested with a sandwich enzyme-linked immunosorbent assay (ELISA) kit following the manufacturer’s protocol (Exbio/Biovendor, Czech Republic). The standard curve measured the concentration of sHLA-G1 and sHLA-G5 isoforms from 3.91 to 125 U/ml. The detection limit of sHLA-G in this assay was 0.6 U/ml.

### Statistical analysis

Statistical analyses concerning sHLA-G concentration measured in the embryo medium were performed using Mann-Whitney or T-student test (GraphPad Prism 5 software). All parameters of these statistical analyses (numbers, medians, means, standard deviation and errors, min, max, and 25-75% percentiles) are part of **Supplementary Tables 2-6**. Values of p < 0.05 were considered significant.

### Ethical approval

Experimental protocols were approved by the Local Ethics Committee (under agreement of the Polish Mothers’ Memorial Hospital–Research Institute in Łódź), and informed consent was obtained from all individual participants included in the study.

## Results

We tested 344 samples of embryo culture supernatants. In 116 samples, no sHLA-G was detected or the result was below the detection limit of the assay. The remaining 228 embryos (66.28%) secreted sHLA-G. Among them, the median secretion was 3.3 U/ml (**Supplementary Table 2**). Arrested development was observed in 206 embryos (59.88%), while 138 (40.12%) had normal development that means they reached blastocyst stage on day 5 or 6 or were transferred earlier.

### Secretion of sHLA-G and the quality of the embryo

**Figure 1:**
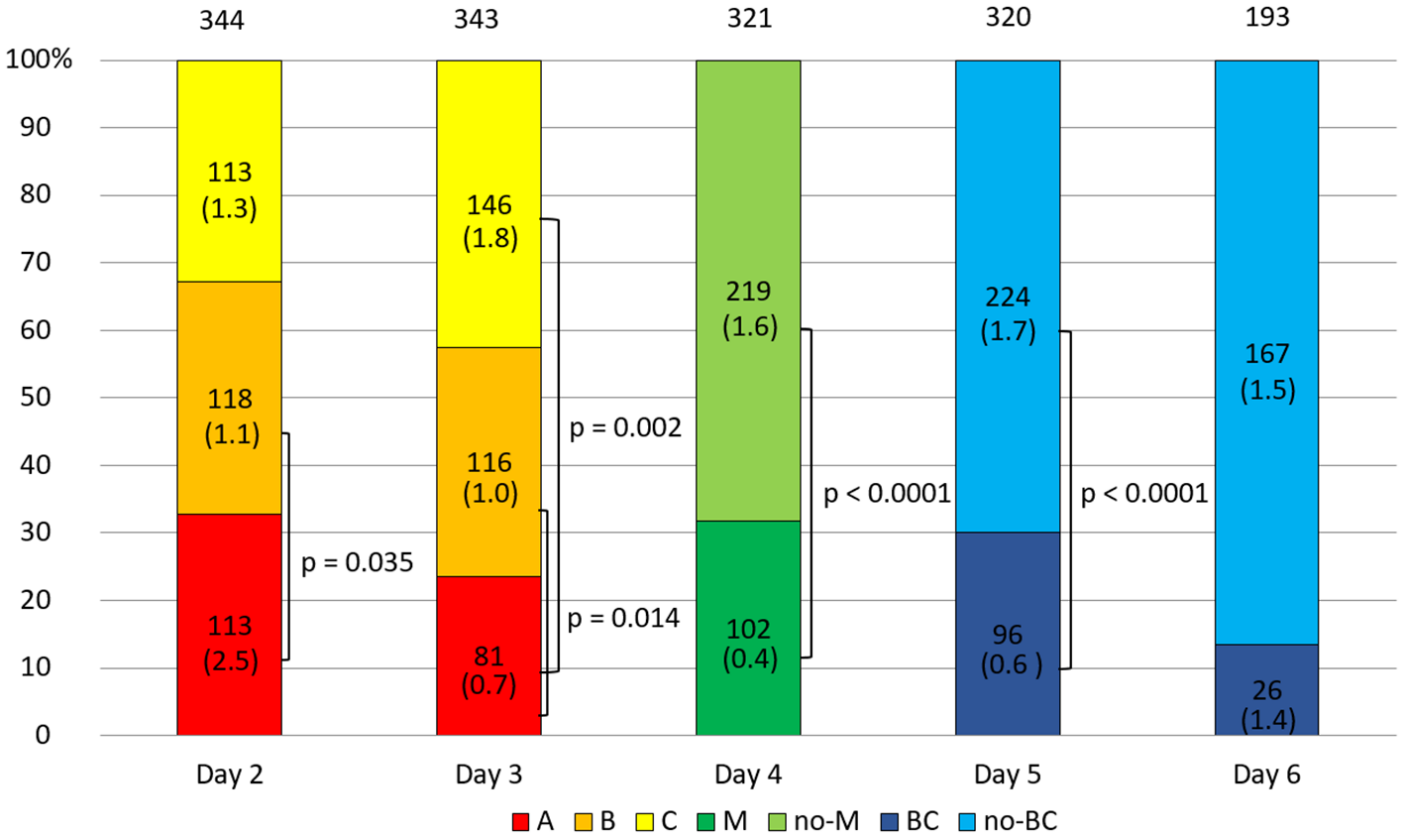
Quality of the embryos depending on the day of development. 1) development of embryos over time from the second to the sixth day (height of the colored stripes); 2) comparisons of secretion between embryos of different classes or stages of development (vertical clamps). A, B, C – class of the embryo; M – embryo in the morula stage; no-M – embryo that has not reached the morula stage; BC – embryo in the blastocyst stage; no-BC – embryo that has not reached the blastocyst stage. Median concentration of sHLA-G was presented in brackets. P-values are calculated by Mann-Whitney test.

In general, the arrested embryos did not differ in sHLA-G secretion from normal developing embryos (**Supplementary Table 2**). In the 2-day embryo group, it was observed that class A embryos secreted more sHLA-G than class B embryos (p = 0.035, **Figure 1**). Through the next day, the percentage of class A embryos decreased in favor of classes B and C. It turned out that on day 3 of culture, class A embryos secreted less sHLA-G than class B (p = 0.014) and C class embryos (p = 0.002, **Figure 1**). In the course of further development, 31.78% of the embryos reached the morula stage and 30.00% of them the blastocyst stage. These embryos secreted less sHLA-G than those that did not obtain these stages (p < 0.0001, **Figure 1**).

### Secretion of sHLA-G by embryo in different ovarian stimulation protocols

**Figure 2:**
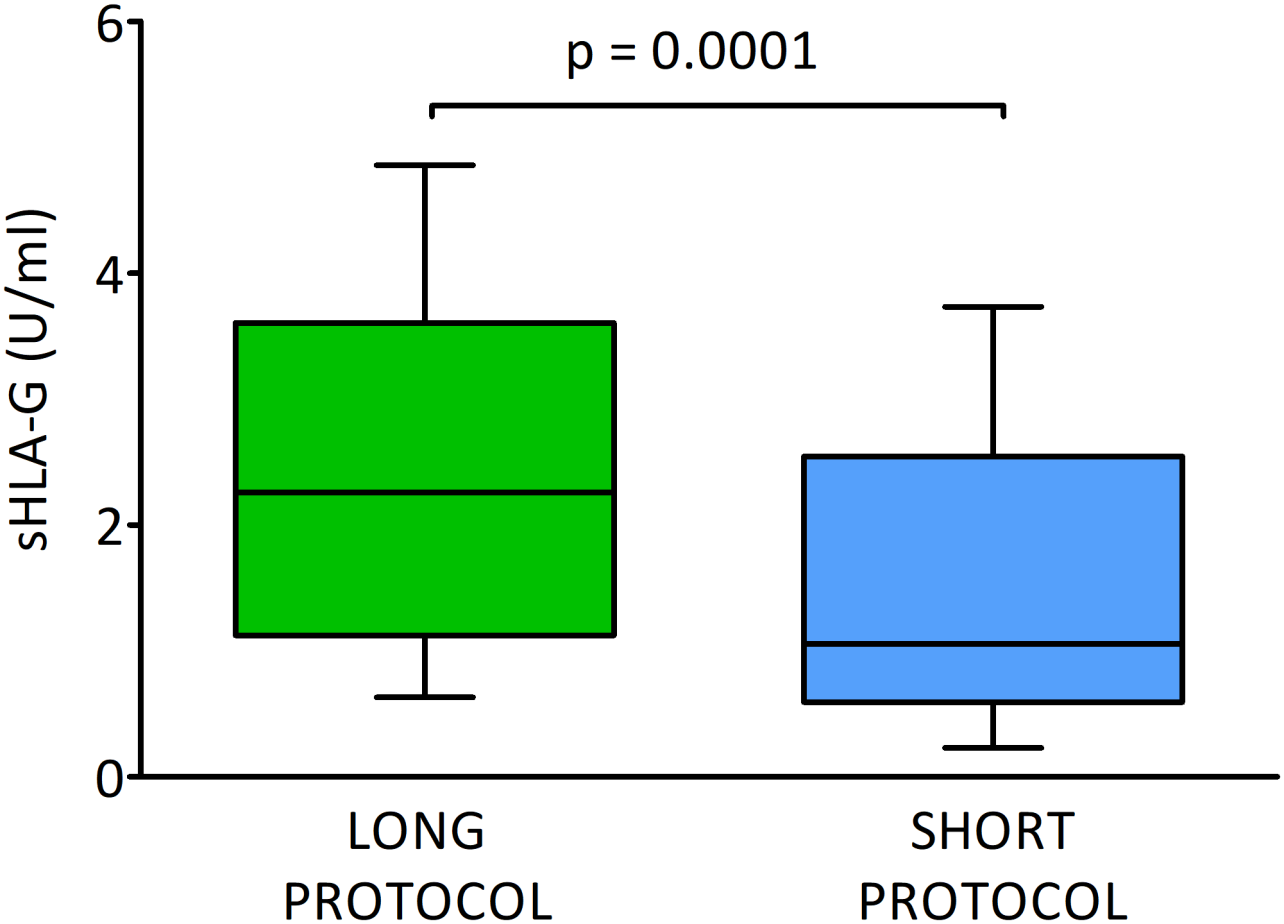
Secretion of soluble HLA-G (U/ml) by embryos in different ovarian stimulation protocols. Green box represents the level of sHLA-G measured in embryo media in long cycle, blue box – in short cycle. Boxes are drawn from the first quartile (25^th^ percentile) to the third quartile (75^th^ percentile). Black lines in boxes are medians. Whiskers represent 10-90 percentiles. P-values are calculated by Mann-Whitney test.

**Figure 3:**
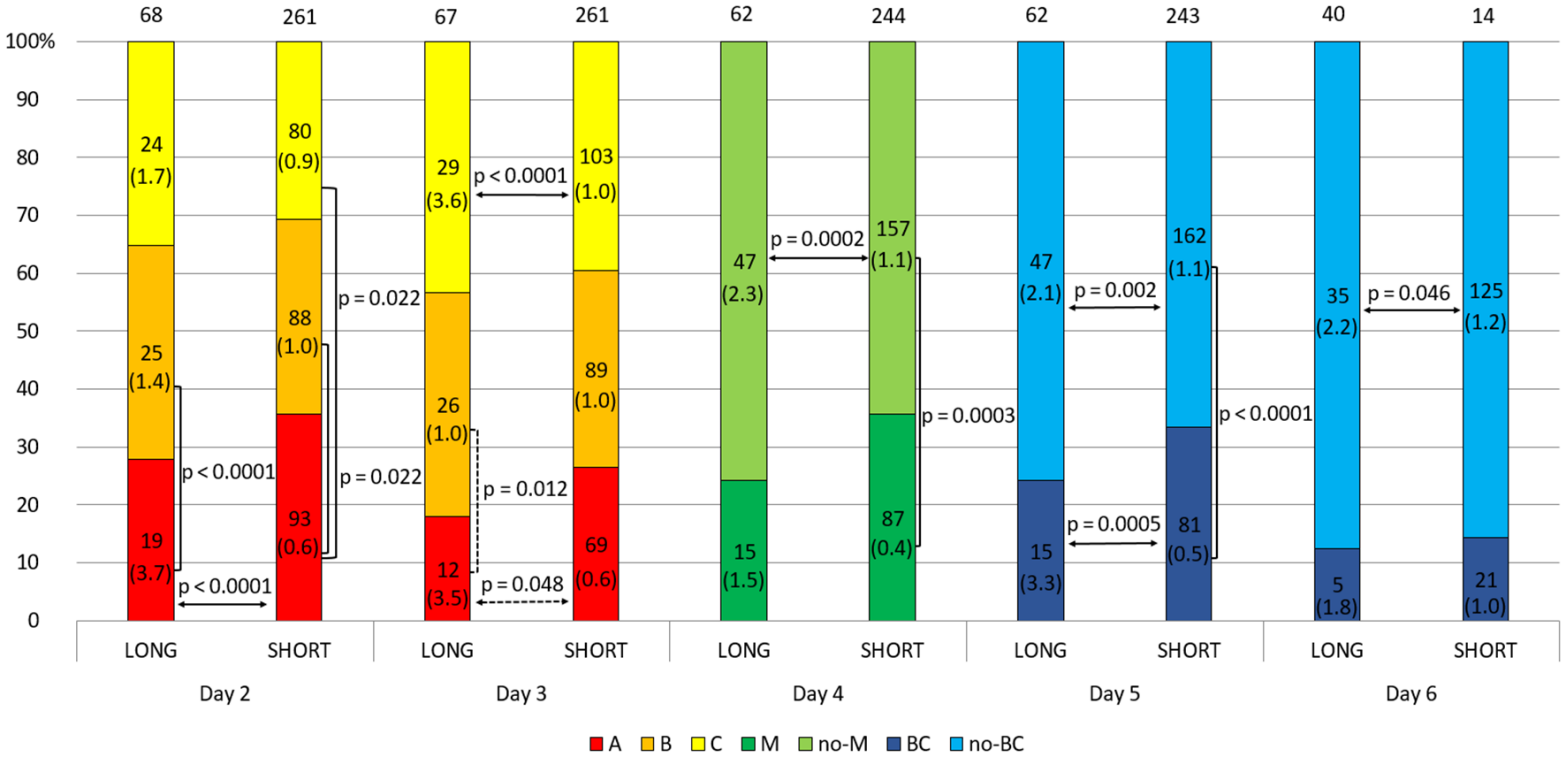
Effect of embryo quality on the concentration of secreted HLA-G in the long and short stimulation protocol. 1) development of embryos over time from the second to the sixth day (height of the colored stripes); 2) secretion comparisons between long and short protocol groups (horizontal arrows); 3) comparisons of secretion between embryos of different classes or stages of development (vertical clamps). A, B, C – class of the embryo, M – embryo in the morula stage; no-M – embryo that has not reached the morula stage; BC – embryo in the blastocyst stage; no-BC – embryo that has not reached the blastocyst stage. Median concentration of sHLA-G was presented in brackets. Dashed line – p-value resulting from analyses with insufficient number of embryos. P-values are calculated by Mann-Whitney test or t-test.

In this section, we consider the hypothesis as to whether the ovarian stimulation protocol has an effect on the quality of the embryo and the level of sHLA-G secreted. We found significant differences between embryos derived from ovarian stimulation according to the short antagonist or long agonist protocol (median 1.1 vs. 2.3 U/ml, p = 0.0001; **Figure 2** and **Supplementary Table 2**). Importantly, this observation was genuine regardless of the day of development and the class of the embryo, so 2- and 3-day embryos of all classes (A-C) from the long protocol elicited higher concentrations of sHLA-G than those from the short stimulation procedure (day 2: median 3.7 vs. 0.6 U/ml, p < 0.0001 for A class embryos; day 3: median 3.5 vs. 0.6 U/ml, p = 0.048 for A class embryos and median 3.6 vs. 1.0 U/ml, p < 0.0001 for C embryos, **Figure 3**). It also did not matter if the embryos reached the morula stage or the blastocyst stage (day 5, p = 0.0005), or did not reach these stages as they developed further (day 4, p = 0.0002, days 5 and 6, p = 0.002, p = 0.046, respectively; **Figure 3**), they secreted more sHLA-G when they came from a long stimulation protocol.

In addition, the analyses show that class A embryos from the second day of development after the long protocol secreted significantly more sHLA-G than class B embryos (day 2: median 3.7 vs. 1.4 U/ml, p < 0.0001, day 3: median 3.5 vs. 1.0 U/ml, p = 0.012; **Figure 3** and **Supplementary Table 3**). Inverse differences can be seen for the embryos after the short protocol (day 2: median 0.6 vs. 1.0 U/ml A vs. B and median 0.6 vs. 0.9 U/ml A vs. C, p = 0.022; day 4: median 0.4 vs. 1.1 U/ml, p = 0.0003; day 5: median 0.5 vs. 1.1 U/ml, p < 0.0001).

It should be noted, however, that a greater percentage of the highest quality embryos originate from the short cycle. This observation is evident on each day of embryo development (more class A embryos, morula, and blastocyst came from a short ovarian stimulation protocol) although the differences are not significant statistically (**Figure 3**).

### Secretion of sHLA-G in fresh and frozen cycles

**Figure 4:**
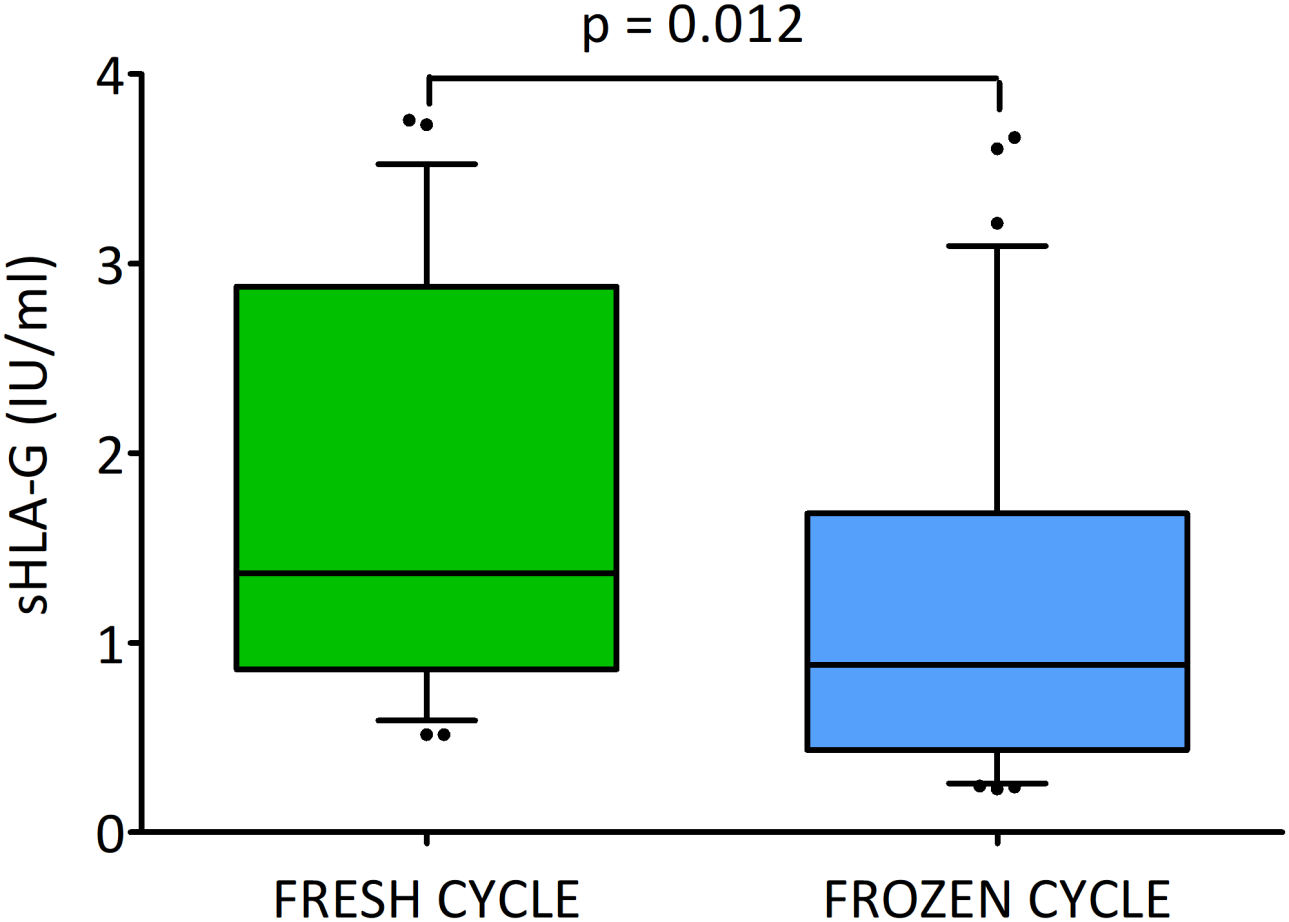
Secretion of soluble HLA-G (U/ml) by embryos in the fresh and frozen cycle. Green box represents the level of sHLA-G measured in embryo media in fresh cycle, blue box – in frozen cycle. Boxes are drawn from the first quartile (25^th^ percentile) to the third quartile (75^th^ percentile). Black lines in boxes are medians. Whiskers represent 10-90 percentiles. P-values are calculated by Mann-Whitney test. Analysis do not include deferred transfers.

When we divided the embryos according to the cycle – fresh or frozen, we observed a statistically significant difference (p = 0.012; **Figure 4**). The embryos transferred in fresh cycles secreted more sHLA-G than the embryos in frozen cycles (median 1.4 vs. 0.9 U/ml, **Supplementary Table 2**).

**Figure 5:**
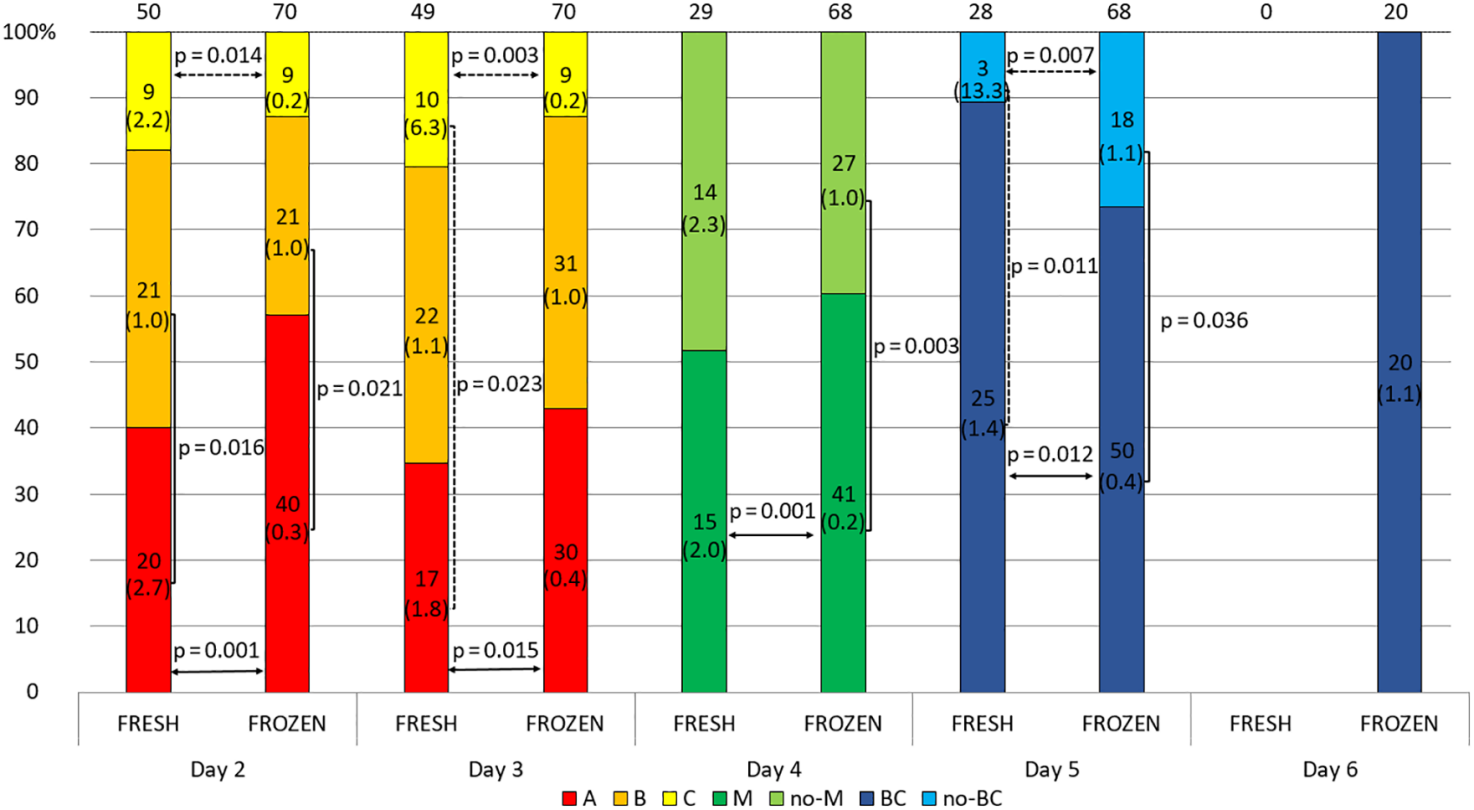
Effect of embryo quality on the concentration of secreted HLA-G in the fresh and frozen cycle. 1) development of embryos over time from the second to the sixth day (height of the colored stripes); 2) secretion comparisons between fresh and frozen cycle groups (horizontal arrows); 3) comparisons of secretion between embryos of different classes or stages of development (vertical clamps). A, B, C – class of the embryo, M – embryo in the morula stage; no-M – embryo that has not reached the morula stage; BC – embryo in the blastocyst stage; no-BC – embryo that has not reached the blastocyst stage; Dashed line – p-value resulting from analyses with insufficient number of embryos. Median concentration of sHLA-G was presented in brackets. P-values are calculated by Mann-Whitney test or t-test.

The blastocysts that were later transferred in the frozen cycle on days 2 and 3 more often obtained class A and rarely class C embryos, and had a higher percentage of morula on day 4 compared to the embryos transferred in the fresh cycle. However, on day 5, reaching the blastocyst stage was more likely among embryos that were later transferred without freezing. Although neither of these differences is statistically reliable, the percentage distribution of embryos in the classes is rather more favorable in the frozen transfer group. After day 5, all fresh transfer embryos were placed in the uterus, therefore no comparison was made for day 6 (**Figure 5** and **Supplementary Table 4**). In general, it can be seen, that embryos which were later destined for fresh transfer expressed higher concentrations of sHLA-G than their counterparts that were frozen after completion of culture. On day 2 class A and C embryos in the fresh cycle produced a higher concentration of sHLA-G than in the frozen cycle (median 2.7 vs. 0.3 U/ml, p = 0.001, 2.2 vs. 0.2 U/ml, p = 0.014, respectively). Similar observation was seen on day 3: median 1.8 vs. 0.4 U/ml, p = 0.015, median 6.3 vs. 0.2 U/ml, p = 0.003, for A and C class embryos, respectively). Also, those that reached the morula and blastocyst stage in the fresh cycle had higher levels of sHLA-G than those in the frozen cycle (day 4: median 2.0 vs. 0.2 U/ml, p = 0.001 and day 5: median 1.4 vs. 0.4 U/ml, p = 0.012, respectively). Embryos, which did not reach the blastocyst stage on day 5 of development elicited more sHLA-G in the fresh cycle (13.3 vs. 1.1 U/ml, p = 0.007; **Figure 5**). **Figure 5** also shows the differences in sHLA-G secretion between embryos of different classes from the 2^nd^ to 5^th^ day of development. Day 2 fresh cycle A class embryos produced more sHLA-G than class B embryos (median 2.7 vs. 1.0 U/ml, p = 0.016). In the case of the frozen cycle, the opposite was true. Class A embryos secrete less sHLA-G than class B embryos (median 0.3 vs. 1.0 U/ml, p = 0.021). Similar observations in the frozen cycle were seen for day 4 (median 0.2 vs. 1.0 U/ml, p = 0.003) and for day 5 (median 0.4 vs. 1.1 U/ml, p = 0.036). In the fresh cycle we also observed that median value of sHLA-G was higher (6.3 vs. 1.8 U/ml) for C class embryos than A (day 3). Analogous trend was seen for embryos that did not reach the blastocyst stage in comparison to the blastocysts (day 5: median 13.3 vs. 1.4 U/ml, p = 0.011).

### Secretion of sHLA-G and pregnancy outcome

**Figure 6:**
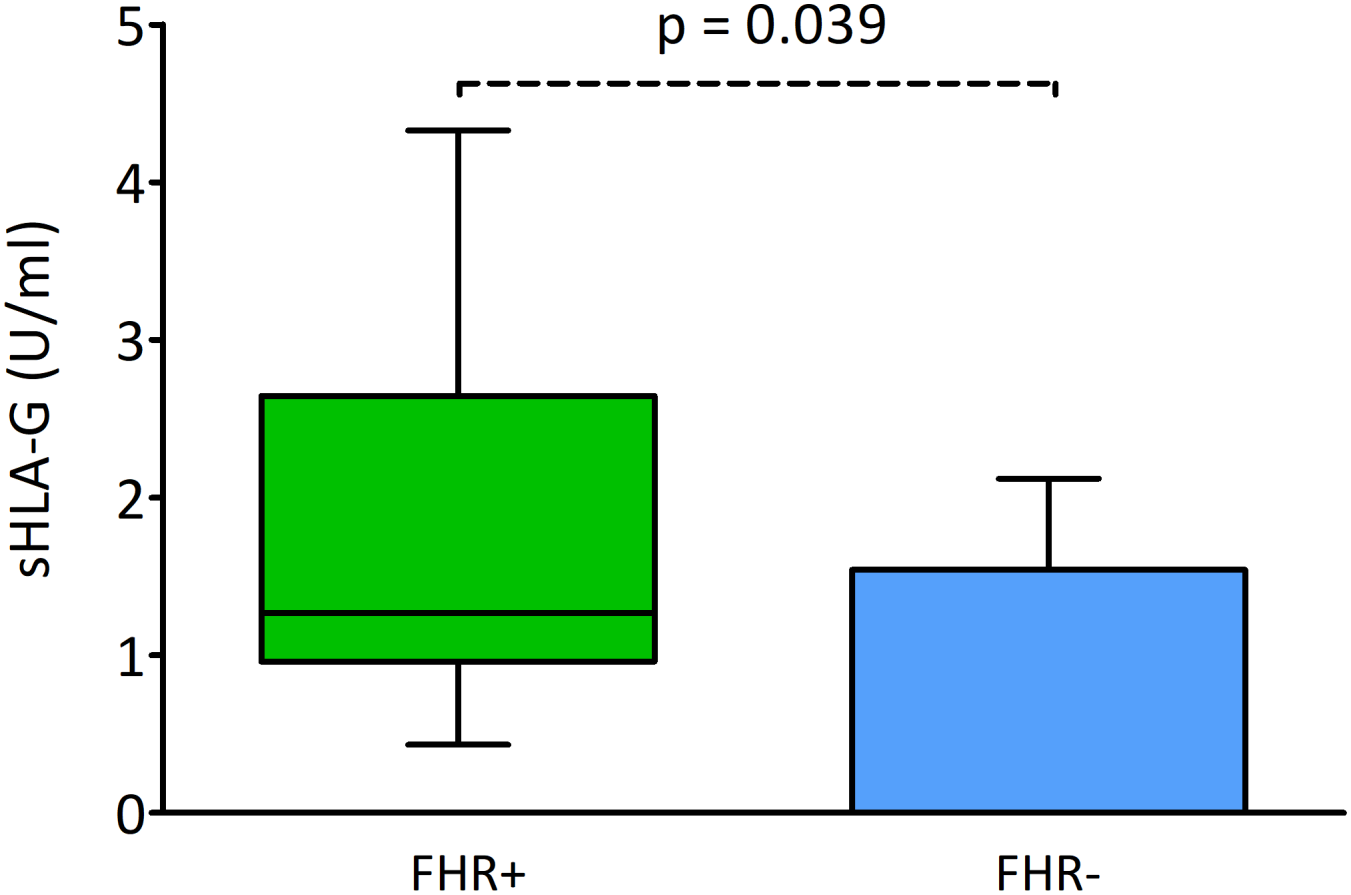
Effect of sHLA-G secretion by embryos on the presence of pregnancy markers. Green box represents the level of sHLA-G measured in embryo media resulting in FHR-positivity; blue box – FHR-negativity. Boxes are drawn from the first quartile (25^th^ percentile) to the third quartile (75^th^ percentile). Black lines in boxes are medians. Whiskers represent 10-90 percentiles. P-values are calculated by Mann-Whitney test. FHR – fetal heart rate.

In this part of the analysis, we examined the level of sHLA-G secreted by the embryos and its potential influence on reproductive success. Embryos whose transfer led to an ongoing pregnancy (FHR-positivity) produced a significantly higher concentration of sHLA-G than embryos whose transfer resulted in FHR-negativity (median 1.27 vs. 0.00 U/ml, p = 0.039; **Figure 6**).

**Figure 7:**
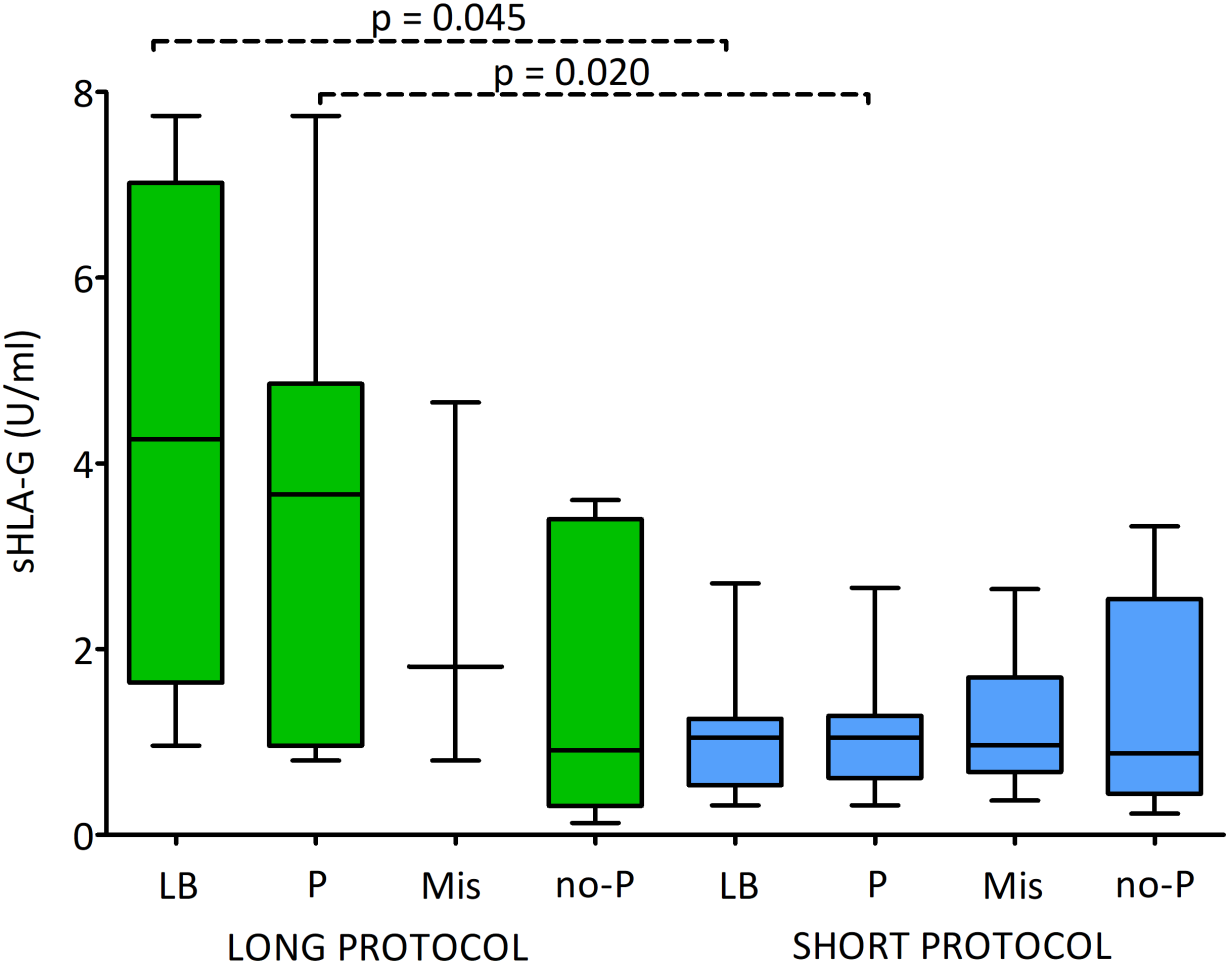
Influence of soluble HLA-G (U/ml) secreted by embryos and long/short cycle on the pregnancy outcome. Green boxes represent the level of sHLA-G measured in embryo media in the long cycle, blue boxes – in the short cycle. Boxes are drawn from the first quartile (25^th^ percentile) to the third quartile (75^th^ percentile). Black lines in boxes are medians. Whiskers represent 10-90 percentiles. P-values are calculated by Mann-Whitney test. Dashed line – p-value resulting from analyses with insufficient number of embryos. P-values are calculated by Mann-Whitney test. LB means live birth, P – pregnancy, MIS – miscarriage, no-P – no pregnancy.

**Figure 7** and **Supplementary Table 5** shows the sHLA-G secretion level by embryos after stimulation with the long or short ovarian stimulation protocol and impact on the fate of the pregnancy. Generally, it can be seen that after the long protocol the level of secretion of sHLA-G is greater than that of the short protocol. But due to the insufficient number of embryos coming from the long protocol, the observed statistical significance obtained (p = 0.045 and p = 0.020) is questionable for interpretation. However, it is worth mentioning that the median values for embryos after the long protocol, which resulted in a live birth, clinical pregnancy, abortion or no pregnancy, decreased from 4.262, 3.665, 1.812 to 0.910 U/ml, respectively. In the case of embryos derived from the short protocol, the medians for live births and clinical pregnancy were the same (1.045 U/ml), while for miscarriage and absence of pregnancy, they decreased by the order of 0.965 to 0.878 U/ml.

**Figure 8:**
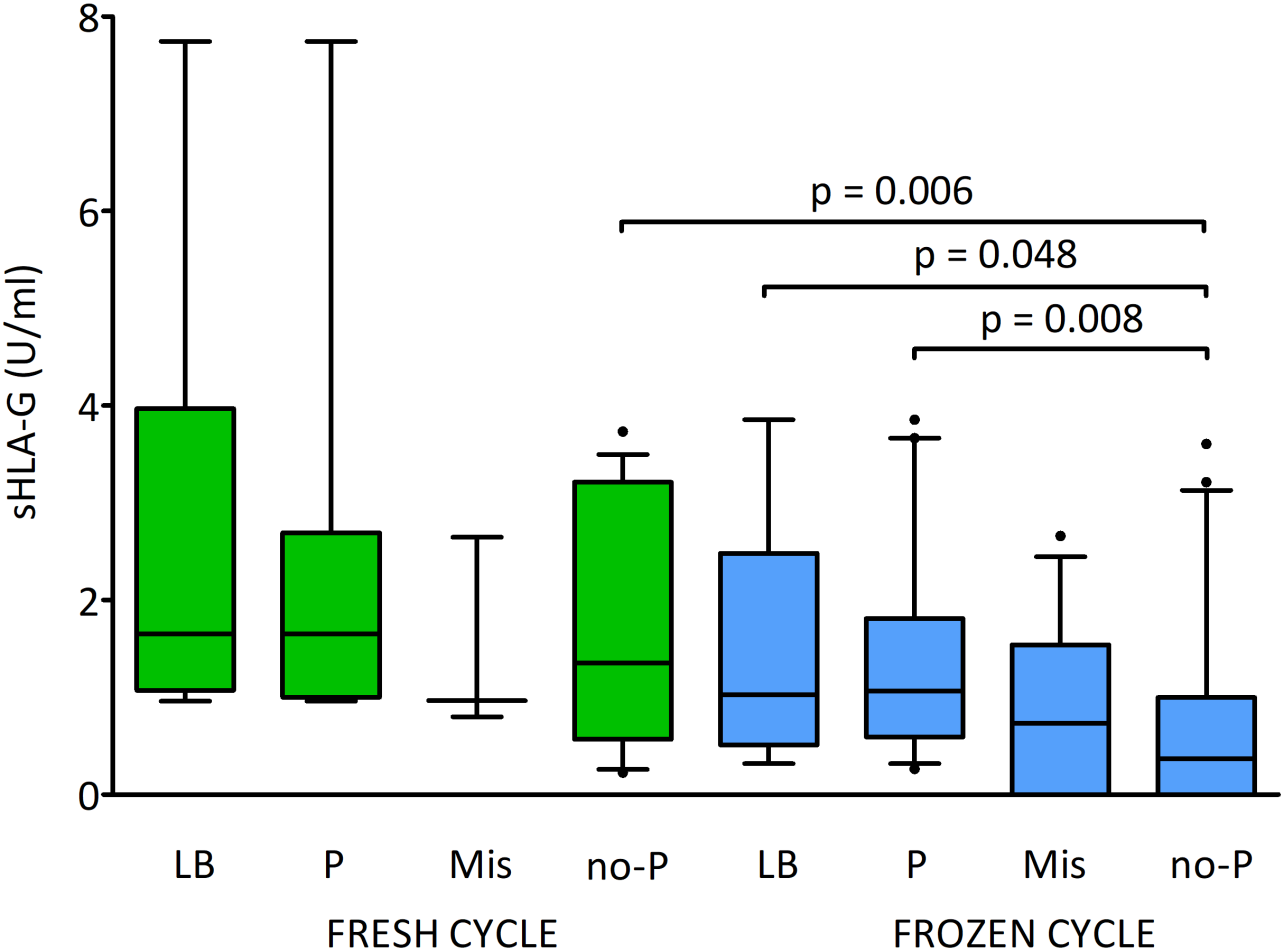
Influence of soluble HLA-G (U/ml) secreted by embryos and fresh/frozen cycle on the pregnancy outcome. Green boxes represent the level of sHLA-G measured in embryo media in fresh cycles, blue boxes – in frozen cycles. Boxes are drawn from the first quartile (25^th^ percentile) to the third quartile (75^th^ percentile). Black lines in boxes are medians. Whiskers represent 10-90 percentiles. P-values are calculated by Mann-Whitney test. LB means live birth, P – pregnancy, MIS – miscarriage, no-P – no pregnancy.

**Figure 8** shows the effect of sHLA-G secretion by the embryo on pregnancy success in fresh and frozen cycles. By classifying the embryos according to whether the pregnancy was live born or if the pregnancy was miscarried, or no pregnancy was detected, we found significantly higher levels of sHLA-G in clinical pregnancy and live birth in comparison to embryos whose transfer ended in no pregnancy (p = 0.008, median 1.1 vs. 0.4 U/ml and p = 0.048, median 1.0 vs. 0.4 U/ml, respectively; **Supplementary Table 6**). These dependencies were visible in the frozen cycles. In fresh cycles, however, they were not statistically significant. In addition, we observed differences in sHLA-G level between the study groups depending on the cycle. Thus, the embryos whose transfer in the fresh cycles did not result in pregnancy secreted more sHLA-G than the embryos in the frozen cycles (p = 0.006, median 1.4 vs. 0.4 U/ml; **Supplementary Table 6**).

## Discussion

In our studies, we detected levels of sHLA-G in embryo culture supernatants to determine: i) whether the secretion of sHLA-G is related to the quality of the embryo; ii) whether the secretion is influenced by the ovarian stimulation protocol; iii) whether sHLA-G secretion could have a potential impact on reproductive success; iv) an influence of sHLA-G secretion on reproductive success in fresh and frozen cycles.

In our research, we found that not all embryos secrete soluble HLA-G. Other researchers also observed that not all embryos were capable of producing sHLA-G (32). However, Criscuoli et al. (18) stated that often, even when no sHLA-G was detected in the culture medium of embryos obtained in the first IVF cycle, secretion of this molecule by embryos from the next IVF cycle of a given couple was observed (18). In addition, such a modulation of HLA-G expression was independent of germline defects. This points to possible epigenetic regulation of the *HLA-G* gene. A defect or deletion of enhancer L which affects the expression of the *HLA-G* could be one of the factors influencing the secretion of sHLA-G (33). The SNP at the position +3142 (rs1063320) of *HLA-G* gene might influence miRNA binding, in which the presence of a guanine increases the affinity for specific microRNAs, such as miR-148a-3p, miR-148b-3p and miR-152, leading to increased mRNA degradation and decreased HLA-G production (34). Moreover, binding of cytosine at position +3142 and miR-365b-5p was impaired by the presence of the 14 bp fragment (rs371194629) (35). In turn, the presence of an adenine at the position +3187 (rs9380142) was associated with decreased mRNA stability, because this variation is close to an AU-rich motif that influences mRNA degradation (36).

Our assumption was that the highest-quality embryos secrete more sHLA-G than lower-quality embryos. Meanwhile, properly developing embryos did not differ in the secretion of this molecule from embryos that stopped development. Literature data reveal that even embryos diagnosed as chromosomal abnormal in preimplantation genetic diagnosis do not differ in sHLA-G secretion from normal ones (25). All these observations point out that the secretion of HLA-G by the developing embryo is not the most important factor for its proper development.

In addition, the relationship between the quality of the embryo and the level of its HLAG secretion is still under discussion. Research by Desai et al. (20), Rebmann et al. (23) and Heidari et al. (37) indicates that the best quality embryos secrete more sHLA-G than the inferior grade embryos (20, 23, 37). These results are in disagreement with the findings in other studies that the presence of sHLA-G in embryo cultures was not related to embryo quality determined morphologically (29, 38, 39). In our study, analyses of only 2-day-old embryos indicate higher sHLA-G secretion by class A embryos. Due to the fact that in the following days of embryo development their quality decreased and the pool of poorer quality embryos (B and C) and those that did not reach the morula and blastocyst stage increased, such an association (i.e., higher sHLA-G concentration in the best quality embryos) was no longer observed.

Some literature data indicated that the long agonist ovarian stimulation protocol is preferable to the short antagonist protocol because of the better pregnancy rates obtained (40–43). According to the authors the long protocol was superior in terms of significantly greater follicle recruitment, rate of oocyte recovery and fertilization, and significantly more embryos available for transfer, as well as clinical pregnancy rates and live birth rates. On the other hand, the meta-analysis by Lambalk et al. (44) comparing the GnRH antagonist with the GnRH agonist protocol in the general population reported no difference in live birth rate (1590 women). However, a significantly lower risk of ovarian hyperstimulation syndrome (OHSS) (22 trials, 5598 women) was found with the use of GnRH antagonists compared to the long GnRH agonist protocol (44). There is also a report where no significant differences were found between the ovarian stimulation protocols used: in the percentage of fertilization, implantation, clinical pregnancies, miscarriages and OHSS (45). This group indicated that satisfactory pregnancy rates were obtained with a short-acting GnRH agonist long protocol and at the same time the costs of this procedure were reduced. Although the first studies reported lower pregnancy rates, several large meta-analyses published in the past years showed similar live birth rates for both protocols. These reports were a background for the latest ESHRE Guidelines (2020) according to which the GnRH antagonist protocol is recommended for predicted normal and high responder as well as PCOS women with regards to improved safety. Only for predicted poor responding women both protocols are equally recommended (46).

Our research indicates that in terms of HLA-G secretion by the embryo the long protocol is more beneficial, than the short protocol. This correlation was shown for the first time. However, we cannot conclude that the ovarian stimulation protocol affects the oocyte quality if more class A embryos were observed after the short stimulation protocol. Also, the percent of obtained blastocysts was higher after short than long protocol. We also did not test the concentration of sHLA-G in the follicular fluid of the ovaries as studied by Rizzo et al. (47) who associated sHLA-G levels in culture supernatants with a fertilized oocyte (47). However, in studies by Jee et al. (38) the follicular fluid sHLA-G level was not related with the formation of good-quality embryo (38).

The chronology of events in IVF procedure indicates that, unlike the type of ovarian stimulation protocol, the type of transfer performed could not have had an effect on HLA-G secretion or the quality of the embryo. However, to understand the impact of the differences between fresh and frozen embryos transferred on reproductive success, we also decided to compare these two groups of embryos. Theoretically, allocation of embryos for transfer in the fresh or frozen cycle should not cause differences in HLA-G secretion between these groups. Surprisingly, our results indicated that the fresh cycle embryos secreted more sHLA-G than the frozen cycle embryos (p = 0.012). Initially, we thought this was due to the fact that the embryologist chose the best quality embryos for fresh transfer to maximize the patient’s chances of getting pregnant. However, the comparison of the quality of embryos in both groups did not confirm this hypothesis. Presumably the reason for the higher HLA-G secretion in the fresh transfer group is the fact that it contains twice as many embryos after a long ovarian stimulation protocol, which secretes statistically significantly higher concentrations of sHLA-G, however, these differences are not statistically reliable.

The insufficient number of transferred embryos coming from the long ovarian stimulation protocol as well as inability to repeat the tests due to the small volume of the embryo culture medium constitutes limitations in our study. On the other hand, the fact that all supernatants came from one clinic speaks in favor of our research, as Tabiasco et al. (26) demonstrated in a meta-analysis the influence of assisted reproductive procedures (ART) on sHLA-G secretion by the embryo. The authors also showed that the concentration of sHLA-G depends on the type of culture medium (26). Different culture media can affect the quality of the embryo and thus the amount of HLA-G expressed. Therefore, at the design stage of our experiment, we decided to collect the medium after the second day of embryo development, and it seems reasonable. The most reliable results were obtained in the analyses of 2-day and 3-day-old embryos, because in the following days many embryos were transferred or frozen, and therefore their further development was not observed. On the other hand, the sHLA-G test would be useful if it could be used in multiple ART clinics. This indicates the necessity to use identical media for embryo culture.

Taking into account the rates of achieved pregnancies and live births, sHLA-G secreted by the embryo may be a factor supporting implantation. The studies of Desai et al. (20) showed that in transfers where at least one embryo selected for embryo transfer was positive for sHLA-G, the pregnancy rate was 64% and the implantation rate per transferred embryo was 38%. In contrast, patients receiving only sHLA-G negative embryos had both a lower pregnancy rate of 36% and a reduced implantation rate (19%) (20). Our results are concordant with this study. Embryos whose transfer results in pregnancy and/or live birth secrete more sHLA-G compared to those whose transfer ends without pregnancy. Other published studies by Sher et al. (16), (17, 48), Yie et al. (19), Vani et al. (39), Niu et al. (49) are also in line with our research (16, 17, 19, 39, 48, 49). Moreover, similar conclusions can be drawn from a multicenter study published by Rebman et al. (28). In this study, the patient’s age, number of embryos transferred, embryo morphology, and sHLA-G level were independent predictors of pregnancy. The morphological scoring system was the best strategy for embryo selection, but sHLA-G can be considered a second parameter if a choice must be made between embryos of the same morphological quality.

When we consider the rates of live births, clinical pregnancies, miscarriages and non-pregnancies following embryo transfers from different ovarian stimulation procedures, we can conclude that in both subgroups (in long and short protocols) sHLA-G is beneficial for pregnancy development and maintenance. It is worth emphasizing, however, that embryos derived from the short protocol, the transfer of which resulted in childbirth, do not differ substantial in terms of sHLA-G secretion from those after stimulation with the long protocol, and whose transfer did not result in pregnancy. This in turn means that although sHLA-G is beneficial for the development of pregnancy, its low level does not determine the fate of the embryo/pregnancy. A similar relationship can be seen for the embryos transferred in the fresh and frozen cycles.

Our research clearly shows that the concentration of sHLA-G secreted by embryos depends on multiple parameters, including: the day of measurement, the amount of collected material, and above all, the procedure of ovarian stimulation. Higher levels of sHLA-G are potentially associated with pregnancy success.

## Conclusions

i) The ovarian stimulation protocol affects the secretion of sHLA-G by the embryo. Long agonist protocol embryos secrete more sHLA-G than short antagonist protocol embryos.
ii) The embryos in the fresh cycle secreted more sHLA-G than the embryos in the frozen cycle. However, this is probably due to twice the percentage of embryos from the long protocol than the short protocol in this group.
iii) Embryos whose transfer results in pregnancy and/or live birth secrete more sHLA-G compared to those whose transfer ends without pregnancy.
iv) The addition of sHLA-G status (measured after 2^nd^ day of embryo development) to traditional embryo morphological criteria can be used to increase the chance of a successful pregnancy.

## Data availability statement

The original contributions presented in the study are included in the article/**Supplementary Material**. Further inquiries can be directed to the corresponding author.

## Ethics statement

The studies involving human participants were reviewed and approved by Bioethical Committee at the Polish Mothers’ Memorial Hospital–Research Institute in Łódź. The patients/participants provided their written informed consent to participate in this study.

## Author contributions

IN conceived and designed the experiments. IN, KP, AW performed the experiments. AT, IN, KP, PR analysed the data. PR, MR, RK contributed to patients and control recruitments. IN, PR and AT wrote the paper. All authors contributed to the article and approved the submitted version.

## Funding

This study was funded by the Polish National Science Centre (grant no. 2014/13/B/NZ5/00273). Open access was covered by Organon, Poland. The funder was not involved in the study design, collection, analysis, interpretation of data, the writing of this article or the decision to submit it for publication.

## Acknowledgments

We would like to express our gratitude to all patients who agreed to participate in our research and made available material for the research.

## Conflict of interest

The authors declare that the research was conducted in the absence of any commercial or financial relationships that could be construed as a potential conflict of interest.

## Publisher’s note

All claims expressed in this article are solely those of the authors and do not necessarily represent those of their affiliated organizations, or those of the publisher, the editors and the reviewers. Any product that may be evaluated in this article, or claim that may be made by its manufacturer, is not guaranteed or endorsed by the publisher.
