# Supplementary material for: The impact of soluble HLA-G in IVF/ICSI embryo culture medium on implantation success
Source: Front Immunol. 2022 Nov 24;13:982518. doi: 10.3389/fimmu.2022.982518 (PMC9730522; doi:10.3389/fimmu.2022.982518)
Supplement: Supplementary file 1 [file Table_1.docx]

**Supplementary Table 1.**  Embryo scoring (day 2-3) and blastocyst scoring system modified from Gardner and Schoolcraft (day 5-6)

| **Time after ICSI** | **Grade** | **Number of blastomeres** | **Fragmentation** | **Multinucleation**  **or vacuolization**  **of blastomeres** | **Blastomere symmetry** |
| --- | --- | --- | --- | --- | --- |
| 43-45 hours (day 2) | A | 4-6 | < 10% | - | even blastomeres |
|  | B | 2, 3, 4  > 6 | 10-25% | - | even blastomeres |
|  | C | 2, 3, 4  > 6 | 25-50% | + | uneven blastomeres |
|  | D | 2, 3, 4  > 6 | > 50% | + | uneven blastomeres |
| 67-69 hours (day 3) | A | ≥ 8 | < 10% | - | even blastomeres |
|  | B | 6-8 | 10-25% | - | > 80%  even blastomeres |
|  | C | 4-6 | 25-50% | + | uneven blastomeres |
|  | D | < 4  lack of cell division (24 h) | > 50% | + | uneven blastomeres |
| **Time after ICSI** | **Grade** | **Stage** | | **Description** | |
| 114-118 hours (day 5)  138-142 hours (day 6) | 1 | early blastocyst | | blastocoel < 50 % of the volume of the embryo | |
|  | 2 | blastocyst | | blastocoel ≥ 50% of the volume of the embryo | |
|  | 3 | full blastocyst | | blastocoel completely fills embryo | |
|  | 4 | expanded blastocyst | | blastocoel larger than that of early embryo, thinner zona | |
|  |  | **Inner cell mass (ICM)** | | **Trophoectoderm grading (TE)** | |
|  | 4AA | tightly packed and many cells | | many cells forming cohesive epithelium | |
|  | 4BB | loosely grouped and several cells | | few cells forming loose epithelium | |
|  | 4CC | very few cells | | very few large cells | |
