# Supplementary material for: The impact of soluble HLA-G in IVF/ICSI embryo culture medium on implantation success
Source: Front Immunol. 2022 Nov 24;13:982518. doi: 10.3389/fimmu.2022.982518 (PMC9730522; doi:10.3389/fimmu.2022.982518)
Supplement: Supplementary file 2 [file Table_2.docx]

**Supplementary Table 2.** General characteristics of the embryos

| **Aspect** | **All embryos** | **sHLA-G secreting embryos** | **Development** | | **Stimulation protocol** | | **Transfer cycle** | | **Live birth** | **Pregnancy** | **Miscarriage** | **No pregnancy** |
| --- | --- | --- | --- | --- | --- | --- | --- | --- | --- | --- | --- | --- |
|  |  |  | **Arrested** | **Normal** | **Long** | **Short** | **Fresh** | **Frozen** |  |  |  |  |
| Number | 344 | 228 | 206 | 138 | 68 | 261 | 50 | 70 | 29 | 54 | 22 | 62 |
| Minimum | 0.000 | 0.370 | 0.000 | 0.000 | 0.000 | 0.000 | 0.000 | 0.000 | 0.000 | 0.000 | 0.000 | 0.000 |
| 25% Percentile | 0.297 | 1.345 | 0.306 | 0.266 | 0.535 | 0.229 | 0.480 | 0.173 | 0.376 | 0.357 | 0.278 | 0.222 |
| Median | 1.336 | 3.314 | 1.775 | 1.141 | 2.257 | 1.059 | 1.367 | 0.884 | 1.109 | 1.110 | 1.083 | 0.879 |
| 75% Percentile | 4.181 | 5.755 | 4.409 | 3.773 | 4.888 | 3.751 | 4.039 | 3.680 | 4.593 | 4.409 | 3.160 | 3.637 |
| Maximum | 97.780 | 97.780 | 97.780 | 53.260 | 43.170 | 97.780 | 19.700 | 53.260 | 53.260 | 53.26 | 13.730 | 21.850 |
| Mean | 4.172 | 6.204 | 4.577 | 3.561 | 4.682 | 3.764 | 3.602 | 3.441 | 6.369 | 4.710 | 2.485 | 2.346 |
| Std. Deviation | 9.211 | 10.740 | 10.370 | 7.110 | 7.324 | 9.693 | 5.012 | 8.621 | 12.880 | 9.894 | 3.384 | 3.689 |
| Std. Error | 0.495 | 0.708 | 0.719 | 0.605 | 0.882 | 0.599 | 0.709 | 1.030 | 2.391 | 1.346 | 0.722 | 0.468 |
| Lower 95% CI of mean | 3.198 | 4.808 | 3.159 | 2.364 | 2.922 | 2.585 | 2.178 | 1.386 | 1.470 | 2.010 | 0.984 | 1.409 |
| Upper 95% CI of mean | 5.146 | 7.600 | 5.994 | 4.758 | 6.441 | 4.944 | 5.027 | 5.497 | 11.270 | 7.411 | 3.986 | 3.282 |
| D'Agostino & Pearson omnibus normality test K^2^ | 449.100 | 282.700 | 288.800 | 163.600 | 66.910 | 369.600 | 28.330 | 101.100 | 36.320 | 69.880 | 21.170 | 73.850 |

**Stimulation protocol – long vs. short:** p = 0.0001;

**Transfer cycle – fresh vs. frozen:** p = 0.012
