# Supplementary material for: The impact of soluble HLA-G in IVF/ICSI embryo culture medium on implantation success
Source: Front Immunol. 2022 Nov 24;13:982518. doi: 10.3389/fimmu.2022.982518 (PMC9730522; doi:10.3389/fimmu.2022.982518)
Supplement: Supplementary file 3 [file Table_3.docx]

**Supplementary Table 3**. General HLA-G secretion characteristics for embryos derived from the long and short stimulation protocol (25-75 percentile)

| **Stimulation protocol** | **Aspect** | **Day 2** | | | **Day 3** | | | **Day 4** | | **Day 5** | | **Day 6** | |
| --- | --- | --- | --- | --- | --- | --- | --- | --- | --- | --- | --- | --- | --- |
|  |  | **A** | **B** | **C** | **A** | **B** | **C** | **M** | **no-M** | **BC** | **no-BC** | **BC** | **no-BC** |
| **Long** | Number of values | 11 | 13 | 13 | 6 | 14 | 14 | 12 | 24 | 9 | 24 | 3 | 18 |
|  | Minimum | 1.812 | 0.373 | 0.417 | 1.812 | 0.242 | 1.231 | 0.000 | 0.883 | 0.439 | 0.770 | 1.650 | 0.630 |
|  | 25% Percentile | 3.329 | 0.964 | 0.757 | 2.237 | 0.406 | 2.199 | 0.000 | 1.149 | 1.171 | 1.149 | 1.650 | 1.086 |
|  | Median | 3.665 | 1.380 | 1.650 | 3.497 | 1.043 | 3.621 | 1.515 | 2.318 | 3.329 | 2.184 | 1.812 | 2.184 |
|  | 75% Percentile | 4.916 | 2.691 | 8.160 | 4.788 | 2.875 | 5.623 | 3.219 | 3.696 | 4.191 | 3.423 | 3.607 | 3.003 |
|  | Maximum | 5.196 | 3.607 | 14.920 | 5.185 | 3.607 | 13.270 | 4.032 | 5.185 | 4.859 | 4.916 | 3.607 | 4.032 |
|  | Mean | 3.815 | 1.756 | 4.283 | 3.504 | 1.528 | 4.507 | 1.688 | 2.582 | 2.821 | 2.378 | 2.356 | 2.161 |
|  | Std. Deviation | 1.115 | 0.988 | 5.107 | 1.292 | 1.218 | 3.317 | 1.595 | 1.441 | 1.609 | 1.273 | 1.086 | 1.128 |
|  | Std. Error | 0.336 | 0.274 | 1.417 | 0.527 | 0.326 | 0.887 | 0.461 | 0.294 | 0.536 | 0.260 | 0.627 | 0.266 |
|  | Lower 95% CI of mean | 3.065 | 1.159 | 1.196 | 2.149 | 0.825 | 2.592 | 0.674 | 1.974 | 1.584 | 1.841 | -0.342 | 1.600 |
|  | Upper 95% CI of mean | 4.564 | 2.353 | 7.369 | 4.860 | 2.231 | 6.422 | 2.701 | 3.191 | 4.058 | 2.916 | 5.054 | 2.721 |
|  | D'Agostino & Pearson  omnibus normality test K^2^ | 0.498 | 1.154 | 4.458 | N too  small | 2.755 | 10.450 | 5.066 | 3.899 | 1.560 | 2.370 | N too  small | 3.377 |
| **Short** | Number of values | 70 | 44 | 41 | 52 | 45 | 53 | 66 | 79 | 61 | 82 | 11 | 64 |
|  | Minimum | 0.000 | 0.266 | 0.302 | 0.000 | 0.227 | 0.245 | 0.000 | 0.302 | 0.000 | 0.245 | 0.245 | 0.132 |
|  | 25% Percentile | 0.000 | 0.671 | 0.576 | 0.000 | 0.446 | 0.628 | 0.000 | 0.595 | 0.000 | 0.580 | 0.310 | 0.417 |
|  | Median | 0.588 | 1.048 | 0.932 | 0.633 | 1.027 | 0.965 | 0.381 | 1.055 | 0.538 | 1.128 | 1.040 | 1.248 |
|  | 75% Percentile | 2.572 | 1.814 | 2.319 | 2.665 | 2.327 | 2.292 | 1.763 | 2.383 | 1.275 | 2.580 | 2.120 | 2.768 |
|  | Maximum | 4.298 | 3.172 | 22.110 | 4.352 | 3.283 | 3.287 | 3.896 | 3.334 | 3.437 | 3.804 | 2.839 | 3.896 |
|  | Mean | 1.176 | 1.305 | 1.854 | 1.307 | 1.345 | 1.369 | 1.031 | 1.441 | 0.876 | 1.529 | 1.285 | 1.592 |
|  | Std. Deviation | 1.372 | 0.856 | 3.385 | 1.522 | 1.049 | 0.924 | 1.300 | 0.978 | 1.048 | 1.102 | 0.956 | 1.229 |
|  | Std. Error | 0.164 | 0.129 | 0.529 | 0.211 | 0.156 | 0.127 | 0.160 | 0.110 | 0.134 | 0.122 | 0.288 | 0.154 |
|  | Lower 95% CI of mean | 0.848 | 1.045 | 0.786 | 0.883 | 1.030 | 1.115 | 0.711 | 1.222 | 0.607 | 1.287 | 0.643 | 1.285 |
|  | Upper 95% CI of mean | 1.503 | 1.565 | 2.922 | 1.731 | 1.661 | 1.624 | 1.350 | 1.660 | 1.144 | 1.771 | 1.927 | 1.899 |
|  | D'Agostino & Pearson  omnibus normality test K^2^ | 13.460 | 6.111 | 85.960 | 8.323 | 8.094 | 9.909 | 10.940 | 16.740 | 12.170 | 28.030 | 1.858 | 27.810 |

**N –** number of embryos; **M** – morula; **no-M** – embryo that has not reached the morula stage; **BC** – blastocyst; **no-BC** – embryo that has not reached the blastocyst stage;

**Long stimulation protocol: Day 2**: A vs. B p < 0.0001; **Day 3**: A vs. B p = 0.012;

**Short stimulation protocol: Day 2:** A vs. B p = 0.022; A vs. C p = 0.022; **Day 4:** M vs. no-M p = 0.0003; **Day 5:** BC vs. no-BC p < 0.0001;

**Stimulation protocol – long vs. short: Day 2:** A vs. A p < 0.0001; **Day 3**: A vs. A p = 0.048; C vs. C p < 0.0001; **Day 4:** no-M vs. no-M p = 0.0002; **Day 5:** BC vs. BC p = 0.0005; no-BC vs. no-BC p = 0.002; **Day 6:** no-BC vs. no-BC p = 0.046;
