# Supplementary material for: The impact of soluble HLA-G in IVF/ICSI embryo culture medium on implantation success
Source: Front Immunol. 2022 Nov 24;13:982518. doi: 10.3389/fimmu.2022.982518 (PMC9730522; doi:10.3389/fimmu.2022.982518)
Supplement: Supplementary file 4 [file Table_4.docx]

**Supplementary Table 4**. General HLA-G secretion characteristics for embryos derived from the fresh and frozen embryo transfer (25-75 percentile)

| **Embryo transfer** | **Aspect** | **Day 2** | | | **Day 3** | | | **Day 4** | | **Day 5** | | **Day 6** |
| --- | --- | --- | --- | --- | --- | --- | --- | --- | --- | --- | --- | --- |
|  |  | **A** | **B** | **C** | **A** | **B** | **C** | **M** | **no-M** | **BC** | **no-BC** | **BC** |
| **Fresh cycle** | Number of values | 10 | 11 | 5 | 10 | 12 | 6 | 8 | 8 | 13 | 3 |  |
|  | Minimum | 0.686 | 0.266 | 0.538 | 0.583 | 0.266 | 2.200 | 0.878 | 0.492 | 0.538 | 7.750 |  |
|  | 25% Percentile | 1.120 | 0.492 | 0.752 | 0.673 | 0.504 | 2.450 | 1.060 | 0.644 | 0.920 | 7.750 |  |
|  | Median | 2.680 | 1.040 | 2.200 | 1.780 | 1.080 | 6.310 | 2.010 | 2.290 | 1.380 | 13.300 |  |
|  | 75% Percentile | 3.360 | 1.380 | 3.130 | 2.860 | 2.450 | 13.400 | 3.170 | 2.740 | 2.590 | 20.500 |  |
|  | Maximum | 5.360 | 3.100 | 3.730 | 3.330 | 3.440 | 13.700 | 3.440 | 7.750 | 3.330 | 20.500 |  |
|  | Mean | 2.600 | 1.240 | 1.990 | 1.830 | 1.400 | 7.400 | 2.090 | 2.460 | 1.740 | 13.900 |  |
|  | Std. Deviation | 1.410 | 0.927 | 1.280 | 1.150 | 1.100 | 5.140 | 1.060 | 2.330 | 0.936 | 6.390 |  |
|  | Std. Error | 0.445 | 0.280 | 0.572 | 0.363 | 0.318 | 2.100 | 0.374 | 0.823 | 0.260 | 3.690 |  |
|  | Lower 95% CI of mean | 1.590 | 0.614 | 0.407 | 1.010 | 0.705 | 2.000 | 1.200 | 0.510 | 1.180 | -2.020 |  |
|  | Upper 95% CI of mean | 3.610 | 1.860 | 3.580 | 2.660 | 2.100 | 12.800 | 2.970 | 4.400 | 2.310 | 29.700 |  |
|  | D'Agostino & Pearson  omnibus normality test K^2^ | 11.900 | 4.150 | N too small | 9.280 | 3.670 | N too small | 16.900 | 5.550 | 18.600 | 0.850 |  |
| **Frozen cycle** | Number of values | 30 | 11 | 7 | 23 | 17 | 7 | 31 | 15 | 38 | 10 | 10 |
|  | Minimum | 0.000 | 0.651 | 0.000 | 0.000 | 0.319 | 0.000 | 0.000 | 0.432 | 0.000 | 0.245 | 0.245 |
|  | 25% Percentile | 0.000 | 0.686 | 0.000 | 0.000 | 0.678 | 0.000 | 0.000 | 0.669 | 0.000 | 0.554 | 0.554 |
|  | Median | 0.341 | 1.030 | 0.240 | 0.432 | 1.030 | 0.240 | 0.240 | 1.040 | 0.436 | 1.120 | 1.120 |
|  | 75% Percentile | 1.220 | 2.120 | 0.439 | 1.270 | 2.850 | 0.439 | 1.060 | 1.930 | 1.090 | 1.980 | 1.980 |
|  | Maximum | 3.670 | 3.610 | 1.280 | 3.670 | 3.730 | 0.879 | 3.670 | 3.610 | 3.900 | 2.660 | 2.660 |
|  | Mean | 0.798 | 1.470 | 0.315 | 0.815 | 1.590 | 0.258 | 0.693 | 1.390 | 0.815 | 1.280 | 1.280 |
|  | Std. Deviation | 1.050 | 0.980 | 0.457 | 1.050 | 1.190 | 0.321 | 1.040 | 0.946 | 1.150 | 0.819 | 0.819 |
|  | Std. Error | 0.191 | 0.295 | 0.173 | 0.218 | 0.288 | 0.121 | 0.186 | 0.244 | 0.187 | 0.259 | 0.259 |
|  | Lower 95% CI of mean | 0.406 | 0.813 | -0.108 | 0.362 | 0.978 | -0.039 | 0.313 | 0.865 | 0.436 | 0.694 | 0.694 |
|  | Upper 95% CI of mean | 1.190 | 2.130 | 0.738 | 1.270 | 2.200 | 0.555 | 1.070 | 1.910 | 1.190 | 1.870 | 1.870 |
|  | D'Agostino & Pearson  omnibus normality test K^2^ | 11.900 | 4.150 | N too small | 9.280 | 3.670 | N too small | 16.900 | 5.550 | 18.600 | 0.850 | 0.850 |

**N –** number of embryos; **M** – morula; **no-M** – embryo that has not reached the morula stage; **BC** – blastocyst; **no-BC** – embryo that has not reached the blastocyst stage;

**Fresh embryo transfer: Day 2**: A vs. B p = 0.016; **Day 3**: A vs. C p = 0.023; **Day 5**: BC vs. no-BC p = 0.011;

**Frozen embryo transfer: Day 2:** A vs. B p = 0.021; **Day 4:** M vs. no-M p = 0.003; **Day 5**: BC vs. no-BC p = 0.036;

**Embryo transfer – fresh vs frozen: Day 2:** A vs. A p = 0.001; C vs. C p = 0.014; **Day 3:** A vs. A p = 0.015; C vs. C p = 0.003; **Day 4:** M vs. M p = 0.001; **Day 5:** BC vs. BC p = 0.012; **Day 5:** no-BC vs. no-BC p = 0.007;
