# Supplementary material for: The impact of soluble HLA-G in IVF/ICSI embryo culture medium on implantation success
Source: Front Immunol. 2022 Nov 24;13:982518. doi: 10.3389/fimmu.2022.982518 (PMC9730522; doi:10.3389/fimmu.2022.982518)
Supplement: Supplementary file 5 [file Table_5.docx]

**Supplementary Table 5.** sHLA-G secretion by embryos after stimulation with the long or short protocol and the impact on the pregnancy fate (25-75 percentile)

| **Stimulation protocol** | **Aspect** | **Live birth** | **Pregnancy** | **Miscarriage** | **No pregnancy** |
| --- | --- | --- | --- | --- | --- |
| **Long** | Number | 4 | 7 | 3 | 6 |
|  | Minimum | 0.961 | 0.802 | 0.802 | 0.130 |
|  | 25% Percentile | 1.637 | 0.961 | 0.802 | 0.312 |
|  | Median | 4.262 | 3.665 | 1.812 | 0.910 |
|  | 75% Percentile | 7.024 | 4.859 | 4.656 | 3.399 |
|  | Maximum | 7.745 | 7.745 | 4.656 | 3.607 |
|  | Mean | 4.308 | 3.500 | 2.423 | 1.543 |
|  | Std. Deviation | 2.813 | 2.510 | 1.998 | 1.553 |
|  | Std. Error | 1.406 | 0.949 | 1.154 | 0.634 |
|  | Lower 95% CI of mean | -0.168 | 1.178 | -2.541 | -0.087 |
|  | Upper 95% CI of mean | 8.783 | 5.822 | 7.388 | 3.173 |
|  | D'Agostino & Pearson omnibus normality test K^2^ | N too small | N too small | N too small | N too small |
| **Short** | Number | 12 | 22 | 9 | 25 |
|  | Minimum | 0.319 | 0.319 | 0.370 | 0.230 |
|  | 25% Percentile | 0.534 | 0.615 | 0.679 | 0.443 |
|  | Median | 1.045 | 1.045 | 0.965 | 0.878 |
|  | 75% Percentile | 1.250 | 1.284 | 1.695 | 2.541 |
|  | Maximum | 2.709 | 2.661 | 2.645 | 3.325 |
|  | Mean | 1.084 | 1.139 | 1.202 | 1.302 |
|  | Std. Deviation | 0.721 | 0.690 | 0.736 | 1.094 |
|  | Std. Error | 0.208 | 0.147 | 0.245 | 0.219 |
|  | Lower 95% CI of mean | 0.626 | 0.833 | 0.636 | 0.850 |
|  | Upper 95% CI of mean | 1.542 | 1.445 | 1.767 | 1.753 |
|  | D'Agostino & Pearson omnibus normality test K^2^ | 5.595 | 5.341 | 3.019 | 4.998 |
