# Supplementary material for: The impact of soluble HLA-G in IVF/ICSI embryo culture medium on implantation success
Source: Front Immunol. 2022 Nov 24;13:982518. doi: 10.3389/fimmu.2022.982518 (PMC9730522; doi:10.3389/fimmu.2022.982518)
Supplement: Supplementary file 6 [file Table_6.docx]

**Supplementary Table 6**. sHLA-G secretion by the embryo on the pregnancy success in the fresh and frozen cycles (25-75 percentile)

| **Embryo transfer** | **Aspect** | **Live birth** | **Pregnancy** | **Miscarriage** | **No pregnancy** |
| --- | --- | --- | --- | --- | --- |
| **Fresh cycle** | Number | 6 | 8 | 3 | 17 |
|  | Minimum | 0.961 | 0.961 | 0.802 | 0.227 |
|  | 25% Percentile | 1.072 | 1.001 | 0.802 | 0.569 |
|  | Median | 1.655 | 1.655 | 0.965 | 1.353 |
|  | 75% Percentile | 3.968 | 2.693 | 2.645 | 3.214 |
|  | Maximum | 7.745 | 7.745 | 2.645 | 3.733 |
|  | Mean | 2.639 | 2.431 | 1.471 | 1.746 |
|  | Std. Deviation | 2.599 | 2.275 | 1.020 | 1.302 |
|  | Std. Error | 1.061 | 0.804 | 0.589 | 0.316 |
|  | Lower 95% CI of mean | -0.088 | 0.529 | -1.064 | 1.076 |
|  | Upper 95% CI of mean | 5.366 | 4.332 | 4.005 | 2.415 |
|  | D'Agostino & Pearson omnibus normality test K^2^ | N too small | 15.150 | N too small | 8.840 |
| **Frozen cycle** | Number | 9 | 19 | 13 | 24 |
|  | Minimum | 0.319 | 0.264 | 0.000 | 0.000 |
|  | 25% Percentile | 0.512 | 0.592 | 0.000 | 0.000 |
|  | Median | 1.027 | 1.063 | 0.735 | 0.366 |
|  | 75% Percentile | 2.481 | 1.812 | 1.541 | 1.000 |
|  | Maximum | 3.855 | 3.855 | 2.661 | 3.607 |
|  | Mean | 1.431 | 1.341 | 0.851 | 0.808 |
|  | Std. Deviation | 1.358 | 1.057 | 0.912 | 1.115 |
|  | Std. Error | 0.453 | 0.243 | 0.253 | 0.228 |
|  | Lower 95% CI of mean | 0.387 | 0.832 | 0.300 | 0.337 |
|  | Upper 95% CI of mean | 2.475 | 1.851 | 1.401 | 1.279 |
|  | D'Agostino & Pearson omnibus normality test K^2^ | 3.957 | 7.732 | 1.688 | 11.400 |

**Frozen cycle:** pregnancy vs. no pregnancy p = 0.008; live birth vs. no pregnancy p = 0.048;

**Transfer cycle – fresh vs. frozen:** no pregnancy vs. no pregnancy p = 0.006
